# Supplementary material for: A cost analysis of implementing mobile health facilitated tuberculosis contact investigation in a low-income setting
Source: PLoS One. 2022 Apr 1;17(4):e0265033. doi: 10.1371/journal.pone.0265033 (PMC8975098; doi:10.1371/journal.pone.0265033)
Supplement: S1 Table — (DOCX) [file pone.0265033.s001.docx]

**Table S1***.* Detailed list of cost components considered for the mHealth-facilitated contact investigation program.

| **Activities** | **Cost component** | **Type of data collected** |
| --- | --- | --- |
| ***Phase I: Development*** |  |  |
| Formative Research  (Feb 2014-May 2014) | Interviews/FGDs | This included the cost of a participation incentive and a transport refund for household contacts, CHWs, and health workers who participated in in-depth-interviews and focus group discussions (FGD). |
|  | Training | Community health workers (CHWs) were trained to conduct HIV testing and counseling, and study staff were trained to use and troubleshoot CommCare, the electronic case record system. |
|  | Staffing | This included salaries for research staff including a social scientist, a laboratory technician, information technology (IT) personnel, and a medical officer/project manager. |
|  | Building operations | The following building operations for the supervision team were costed: security (not personnel), electricity, water, phone, internet, general waste removal and cleaning services. |
| Software Customization  (May 2014-Sept 2015) | Staff | The staff salary costs included: IT personnel, IT consultant, a medical officer, and a laboratory technologist. |
|  | Software | These included the annual subscription cost of the CommCare electronic case record software and the purchase costs for the biometric fingerprint identification system software. |
|  | Building operations | These included security (not personnel), electricity, water, phone, internet, general waste removal and cleaning services. |
|  | Equipment | These included tablets, tablet accessories, fingerprint scanners, and laptops. |
| mHealth TB Contact Investigation Pilot  (Sept 2015-July 2016) | Staff | These included IT personnel, an IT consultant, a medical officer, a laboratory technologist, a data manager, and CHWs. |
|  | Building operations (Supervision) | The following building operations for the supervision team were costed: security (not personnel), electricity, water, phone, internet, general waste removal and cleaning services. |
|  | Building operation  (Patient Care) | The following building operations for patient care were costed: security (not personnel), electricity, water, phone, internet, general waste removal specialized waste removal and cleaning services. |
|  | Vehicles | This included the purchase of a vehicle for supervision purposes. |
|  | Supplies and Other Resources | The following consumables were costed for HIV testing and sputum collection: alcohol swabs, bio-hazard bags (21 inch), blue or black pens, 200g hospital-quality cotton wool, Determine HIV-1/2 test kits, 4 mL vacutainer tubes with ethylenediaminetetraacetic acid (EDTA), disposable non-sterile gloves, lancets - fixed point, lancets - spring loaded, sharps container, StatPak HIV-1/2 test kits, timers, sputum containers, paper towels, permanent sharpie markers, sputum shipping containers, theatre-style hospital masks, internet data bundles, and voice call plans (monthly). |
|  | Training | This included the cost of CHW retraining and continuing education activities. |

***Supplementary Table 1, continued***

| **Activities** | **Cost component** | **Type of data collected** |  |
| --- | --- | --- | --- |
| ***Phase II: Implementation*** |  |  | |
| mHealth TB Contact Investigation Intervention Randomized Controlled Trial  (July 2016-July 2017) | Software | The software cost during this phase included a CommCare annual subscription. | |
|  | Staffing | The included the salaries for IT personnel, an IT consultant, a medical officer, a laboratory technologist, a data manager, and CHWs. | |
|  | Building operations (Supervision) | The following building operations for the supervision team were costed: security (not personnel), electricity, water, phone, internet, general waste removal and cleaning services. | |
|  | Building operations (Patient Care) | The following building operations for the supervision team were costed: security (not personnel), electricity, water, phone, internet, general waste removal specialized waste removal and cleaning services. | |
|  | Consumables | The following consumables were costed for HIV testing and sputum collection: alcohol swabs, bio-hazard bags (21 inch), blue or black pens, 200g hospital-quality cotton wool, Determine HIV-1/2 test kits, 4 mL vacutainer tubes with EDTA, disposable non-sterile gloves, lancets - fixed point, lancets - spring loaded, sharps containers, StatPak HIV-1/2 test kits, timers, sputum containers, paper towels, permanent sharpie markers, sputum shipping containers, theatre-style hospital masks, internet data bundles, and voice call plans (monthly). | |
