# Supplementary material for: A cost analysis of implementing mobile health facilitated tuberculosis contact investigation in a low-income setting
Source: PLoS One. 2022 Apr 1;17(4):e0265033. doi: 10.1371/journal.pone.0265033 (PMC8975098; doi:10.1371/journal.pone.0265033)
Supplement: S2 Table — (DOCX) [file pone.0265033.s002.docx]

**Table S2***.* Community health worker activities identified during time-and-motion surveys.

| **Activity category** | **Activity type** | **Activity description** |
| --- | --- | --- |
| **Clinic activities** | TB patient recruitment | Community health workers (CHW) educated patients about the risks of TB disease and transmission, introduced TB contact investigation, and interviewed index TB patients and enumerated contacts. |
|  | Waiting for clients | CHWs waited at the clinic to enroll new TB index patients. They helped complete patient registers, coordinate TB care with the HIV clinic, and transported specimens to the laboratory for testing. |
|  | Contact evaluation | CHWs received household contacts referred for evaluation at the clinic and helped expedite their care between each of the service points. |
|  | Other | CHWs had time allotted for convenience breaks, tea and lunch breaks, and other clinic activities unrelated to contact investigation. |
| **Community activities** | Travel | CHWs traveled to the community for a household visit and back to the clinic, on foot or using public transportation. |
|  | TB education and counselling | CHWs taught contacts encountered in the household about the risks of TB its transmission and the purpose of TB contact investigation. |
|  | Contact screening | CHWs recruited and enrolled contacts and screened them for TB symptoms and other indications for further TB evaluation. |
|  | HIV testing | CHWs offered fingerprick HIV testing services to all contacts who consented and were ≥15 years of age (for HIV testing activities conducted separately from sputum collection). |
|  | Sputum collection & HIV testing | CHWs collected sputum from symptomatic contacts while awaiting the results of HIV testing (for activities of HIV testing and sputum collection conducted concurrently). |
|  | Sputum collection | CHWs collected sputum sampled at home from all contacts who had one or more symptoms of TB and were ≥5 years old (for activities of sputum collection conducted separately from HIV testing). |
|  | Phone number confirmation | Phone contacts were confirmed using automated short-messaging service (SMS) verification codes, to ensure text messages would be sent to the correct phone number. |
