# Supplementary material for: A cost analysis of implementing mobile health facilitated tuberculosis contact investigation in a low-income setting
Source: PLoS One. 2022 Apr 1;17(4):e0265033. doi: 10.1371/journal.pone.0265033 (PMC8975098; doi:10.1371/journal.pone.0265033)
Supplement: S3 Table — (DOCX) [file pone.0265033.s003.docx]

**Table S3***.* Distribution of community health worker activity time by health center per household TB contact investigated.

| **CHW activities** | | **Median person-minutes**  **per**  **TB household contact investigated**  **(Interquartile range, IQR)** | | | | | | | **Median person minutes per household TB contact investigated** |
| --- | --- | --- | --- | --- | --- | --- | --- | --- | --- |
|  | |  | | | | | | | **(IQR)** |
| **Health Centre** | | **Naguru** | **Kawaala** | **Kisenyi** | **Kisugu** | **Kiswa** | **Kitebi** | **Komamboga** |  |
| **(Number of CHWs)** | | **(n=1)** | **(n=3)** | **(n=3)** | **(n=1)** | **(n=2)** | **(n=2)** | **(n=2)** |  |
| **Clinic activities** | TB patient recruitment | 10 (7-23) | 16 (10-21) | 24 (4-33) | 22 (14-29) | 3 (2-15) | 12 (2-18) | 17 (11-23) | 12 (5-26) |
|  | Waiting for clients | 21 (10-47) | 21 (10-31) | 21 (11-40) | 25 (20-30) | 47 (35-57) | 29 (11-44) | 10 (10-13) | 21 (10-42) |
|  | Contact evaluation | 53 (32-70) | 30 (30-30) | 117 (117-117) | - | - | - | 15 (15-15) | 30 (30-70) |
|  | Other | 12 (11-20) | 10 (3-19) | 7 (5-9) | - | 22 (21-37) | 9 (7-11) | 5 (5-5) | 10 (5-21) |
| **Community activities** | Travel | 20 (15-31) | 10 (9-16) | 26 (21-33) | 21 (21-21) | 21 (16-32) | 20 (10-42) | 19 (15-22) | 21 (14-32) |
|  | TB education & counselling | 9 (7-47) | 20 (12-28) | 7 (5-8) | 5 (5-5) | 28 (28-28) | 16 (16-16) | 22 (22-22) | 7 (5-16) |
|  | Contact screening | 20 (20-30) | 20 (5-30) | 38 (31-50) | 6 (6-8) | 15 (5-18) | 44 (23-53) | - | 20 (11-38) |
|  | HIV testing | 13 (9-15) | 30 (30-45) | 27 (21-49) | - | 305 (305-305) | 28 (28-28) | - | 28 (17- 43) |
|  | Sputum collection & HIV testing | 302 (302-302) | 19 (5-30) | 41 (21--89) | 17 (17-17) | 7 (5-23) | 3 (3-3) | - | 19 (5-30) |
|  | Sputum collection | 92 (60-123) | 30 (30-30) | 25 (25-25) | - | - | 20 (20-20) | - | 29 (25-30) |
|  | Phone number confirmation | - | 10 (8-12) | - | - | - | 20 (20-20) | - | 13 (9-16) |
| **Overall median person-minutes per household contact investigated** | | | | | | | | | **209 (136-362)** |
